# Influence of HFD-induced precocious puberty on neurodevelopment in mice

**DOI:** 10.1186/s12986-021-00604-w

**Published:** 2021-09-16

**Authors:** Tingbei Bo, Jing Wen, Wenting Gao, Liqiu Tang, Min Liu, Dehua Wang

**Affiliations:** 1 State Key Laboratory of Integrated Management of Pest Insects and Rodents, Institute of Zoology, Chinese Academy of Sciences, Beijing, 100101 China; 2 School of Life Science, Shandong University, Qingdao, 266237 China; 3 CAS Center for Excellence in Biotic Interactions, University of Chinese Academy of Sciences, Beijing, 100049 China; 4 College of Life and Environmental Science, Wenzhou University, Wenzhou, 325035 China

**Keywords:** Precocious puberty, Neurodevelopment, High fat diet, Behaviors, Memory

## Abstract

**Background:**

Precocious puberty is frequently associated with obesity, which will lead to long-term effects, especially on growth and reproduction. However, the effect of precocious puberty on children's neurodevelopment is still unknown.

**Objectives:**

Here we evaluated the effect of High fat diet (HFD)-induced precocious puberty on neurodevelopment and behaviors of animals.

**Methods:**

Ovaries sections were stained with hematoxylin–eosin (H&E) using standard techniques. Behavioral tests included elevated plus maze (EPM), open field exploration, Y-Maze, marble burying test, and novelty- suppressed feeding. The expression of genes related to puberty and neural development was detected by immunohistochemistry and Western blot.

**Results:**

Our results showed HFD-induced precocious puberty increased the risk-taking behavior and decreased memory of mice. The content of Tyrosine hydroxylase (TH) and Arginine vasopressin (AVP) in hypothalamus were higher in HFD group than control group. Although the recovery of normal diet will gradually restore the body fat and other physiological index of mice, the anxiety increases in adult mice, and the memory is also damaged.

**Conclusions:**

These findings describe the sensitivity of mice brain to HFD-induced precocious puberty and the irrecoverability of neural damage caused by precocious puberty. Therefore, avoiding HFD in childhood is important to prevent precocious puberty and neurodevelopmental impairment in mice.

**Graphic abstract:**

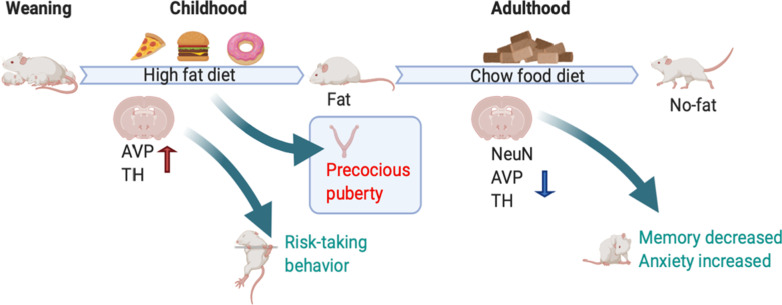

## Background

Puberty is a period in which mammals develop second sex characteristics, accelerate linear growth and gain reproductive capacity [1]. Puberty is mainly regulated by the central nervous system, which was called hypothalamus-pituitary–gonadal axis (HPG axis) [2]. With the improvement of living standards, the age of human puberty is also gradually advanced, many children suffer from precocious puberty. Precocious puberty can lead to rapid bone development, obesity, short stature and increase risk of diabetes and cancer, as well as some psychological problems, including anxiety, depression and social disorder [3, 4]. In addition, early puberty is also associated with higher disease risk in adulthood [4], such as cardiovascular and metabolic diseases, higher susceptibility to cognitive and behavioral disorders, and even reduced life expectancy [5].

Studies have shown that obesity and precocious puberty are inseparable [6], and the increase of fat accumulation will promote precocious puberty in children [7]. Recent evidence suggests that there is a link between an increase in childhood obesity and an earlier age of puberty, especially in girls [7, 8]. In recent years, there have been a lot of studies on the activation and metabolism of neurohormones in puberty [6, 9]. The increase of GnRH neurosecretory activity means the beginning of puberty [10]. Previous studies have found kisspeptin (kiss1) expressed in anteroventral periventricular nucleus and arcuate nucleus neurons acts as a key upstream regulator of the HPG axis in rodents and human [11, 12]. In addition, some studies have shown that kisspeptin and its receptor GPR54 (kiss1r) are expressed in different types of tissues and regulate reproduction directly or indirectly [13]. Kisspeptin is also a key factor in promoting precocious puberty, because the level of kisspeptin in obese precocious puberty children is high [14]; and kiss-1receptor gene over-expression can lead to precocious puberty [15].

Age has a great influence on the behavior of mice, in adolescence, the level of pursuit of novelty increased, more risk-taking behavior [16]. Additionally, the effect of age on behavior is related to sex hormone, for example progesterone [17]. However, the effect of precocious puberty on behavior and neurodevelopment were still unclear, and the impact on adulthood is also rarely studied. High fat diet (HFD) can affect both obesity and neurogenesis [18], like one day after feeding HFD, the episodic memory and spatial memory of mice will be damaged [19]. Ullah et al. [20] established HFD-induced precocious puberty model by feeding HFD (lactation and weaning) for a long time in mice, and found that HFD can cause premature puberty in pups and cause kisspeptin changed in the hypothalamus. Our aim is to establish a HFD-induced precocious puberty model to further explore the effect on neurodevelopment and their behavior. We measured the behavior from many aspects, including memory, anxiety and risk-taking, to fully show the impact of precocious puberty on behavior. At the same time, measure the AVP, TH, Brain-derived neurotrophic factor(BDNF) and Neuronal Nuclei (NeuN) in hypothalamus to explain the brain basis of behavior [21]. Whether the HFD- induced precocious puberty affect the behavior and neurodevelopment in adulthood, and whether the change of diet can make up for the harm of excessive nutrition in childhood were also the direction we explored.

## Materials and methods

### Animals and experimental design

All animals were licensed under the Animal Care and Use Committee of the Institute of Zoology, the Chinese Academy of Sciences. BALC/c mice (21 days old) was bought from SPF (Beijing) Biotechnology Co., Ltd. Mice were housed individually in plastic cages (29 × 18 ×  16 cm^3^), and were maintained at the room temperature of 23 ± 1 °C, under a photoperiod of 16L:8D. During the test, food and water were provided ad libitum. The animal experiments approved by a regulatory institution and performed according to established guidelines. In this study, we used the following two kinds of food:

- Chow food: Fat was 6.2%, carbohydrate was 35.6%, protein was 20.8% and the calorific value was 17.6 kJ/g (Beijing Keao Xieli Feed Co.)
- High-fat food: Fat was 60%, carbohydrate was 20%, protein was 20% and the calorific value was 22.0 kJ/g (Research Diets Inc., D12492, USA.)
- Experimental design 1: To verify that high-fat foods can cause precocious puberty and behavioral changes, 18 female 21 days-old mice were fed by chow food, 18 female 21 days-old mice were fed by high-fat food. When all the mice were puberty (about 36 days-old), 6 mice in each group were randomly sacrificed (n = 6, named HFD and CFD). The rest of the animals were used to measure behavior.
- Experimental design 2: To verify that the change of behavior is not caused by high-fat food, but by precocious puberty, 24 female 21 days-old mice were fed by chow food. 12 of them were injected estradiol (0.5ug/g.day) for 7 days, we called E2 group and another 12 were injected with the placebo (solvents for estradiol) called CON group. 6 mice in each group were randomly sacrificed (n = 6), and the other 6 were for behavior test.
- Experimental design 3: To explore whether the behavioral and neural changes caused by precocious puberty can be reversed. 12 female 21 days-old mice were fed by chow food, 12 female mice were fed by high-fat food, after puberty, two groups were fed with chow food for 60 days, until mice reached full maturity then test behaviors. 6 mice in each group were randomly sacrificed (n = 6, named HF-C, and CF-C), and the other 6 were for behavior test.

### Observation on the state of vulva

Fix the female mice and lift their tails to expose the vulva. Observe the opening of vulva, the color of mucous membrane and the swelling of vulva. Before estrus: vaginal orifice not open, slightly moist, mucous membrane light red, pudendal fold wall slightly swollen. Estrus: vaginal opening, moist, mucus secretion, mucous membrane crinkle wall swelling.

### Vaginal exfoliated cell smear

When the symptoms of estrus appeared, combined with vaginal exfoliated cell smear, we determined that the animal was puberty. Fixed the mice, exposed the vaginal orifice, put a 20ul saline into the vaginal, gently rinse and repeatedly suck for three times. Drop the absorbed liquid on the slide, spread it out and dry naturally. Methanol was fixed for 3 min, dried naturally and stained by Papanicolaou. The nucleus is blue purple, the cytoplasm of non-keratinocytes is light blue, and the cytoplasm of keratinocytes is pink. During the estrus, there are more keratinocytes, nuclear deep into dense.

### Elevated plus maze (EPM)

The EPM raised 90 cm above the ground was formed by a plastic structure in the shape of a cross with four arms (50 cm × 10 cm). The north and south arms were opened, while the east and west arms were enclosed by 36-cm-high walls. The mouse placed on the central platform was allowed to explore the maze for 5 min. The total time spent and the distance traveled in the open and close arms was measured [22].

### Open field exploration

The open field test is widely used to measure anxiety-like in rodents [23, 24]. Mice were placed in the unfamiliar arena (60 × 60 × 45 cm^3^) for 5 min to adapt the environment. After that the activity of the mice were recorded by an overhead video camera for 10 min. We calculated the moving distance in center and the number of times they entered the center. The apparatus for this behavioral test was cleaned thoroughly with 75% ethanol between uses to remove odor cues from previous individuals.

### Y-Maze

Y-Mazes widely used to measure memory ability in rodents [21]. The apparatus consisted of a white plastic maze with three arms (40 cm long, 30 cm high, and 8 cm wide) that intersect at 120°. The three arms are “beginning arm” “food arm” and “novel arm”, and back wall of each arm was marked with different colored shapes. The mice began the test after a 12-h fasting period. First, we only opened “beginning arm” and “food arm”. Mice were placed in for 5 min to adapt and learn the location of the food and association with a colored shape marker. Mice were then removed from the Y-maze for 1 h. Next, in the formal test, we removed the food from “food arm” and opened the “novel arm”. Mice were allowed to move freely for 10 min, and we calculated the distance traveled and amount of time spent in the “food arm”. This test can tell us whether the animal could find the original location of the food using only the markings on the wall.

### Marble burying test

Marble burying test measures the propensity of mice to engage in a digging behavior and is increased in models of anxiety [25]. Cages (42 × 27 × 20 cm^3^) were filled with 5 cm of bedding material and on top of the bedding material, 20 blue glass marbles were arranged in an equidistant 5 × 4 grid and the animals were given access to the marbles for 30 min. After the test, the mice were gently removed from the cage. Marbles covered for more than 50% by bedding were scored as buried, what need to measured is the number of buried marbles.

### Novelty- suppressed feeding

The novelty-suppressed feeding test has also been validated as a test that is sensitive to anxiety-related behavior [26]. The novelty-suppressed feeding test apparatus consisted of a plastic arena (60 ×  60 ×  40 cm^3^). A single food pellet was placed on a piece of white plate (9 cm in diameter) positioned in the center of the arena. Mice were deprived of food in their home cages for 24 h before test. We recorded the amount of time spent before the mouse approached the pellet and began feeding [27].

### Hormone determination

Serum estradiol concentrations were quantified using an 17 beta Estradiol ELISA kit (ab 108664, abcam) according to the instructions. The minimum detected concentration of the kit was 8.68 pg/ml for EST.

Serum leptin concentrations were quantified using an Leptin mouse Elisa kit (ab 100718, Abcam) according to the instructions. The minimum detected concentration of the kit was 4 pg/mL for leptin.

Serum luteinizing hormone (LH) concentrations and follicle stimulating hormone (FSH) concentrations were quantified using LH mouse Elisa kit (AB-C4424B, Abmart) and FSH mouse Elisa kit (AB-3291A, Abmart) according to the instructions.

### Western blot

Hypothalamus were homogenized in RIPA buffer and cleared by centrifugation, according to the standard techniques [28]. Western blots of whole tissue lysates were probed with primary antibodies against Kisspeptin1 (KISS1, Solarbio, K009431P), Kisspeptin1 receptor (KISS1R, Solarbio, K003544P), GnRH(PA5-97047, Thermo Fisher), GnRH receptor (abcam, ab183079), Tyrosine hydroxylase (TH, AB152; Merck Millipore), Arginine vasopressin (AVP, AB1565, Merck Millipore), Neuronal Nuclei (NeuN; MAB377, Merck Millipore), brain-derived neurotrophic factor (BDNF; AB203573, Abcam) and β-Tubulin (A01030HRP, Abbkine). The secondary antibody used was either peroxidase- conjugated goat anti-rabbit IgG (111-035-003; Jackson), or peroxidase-conjugated goat anti-mice IgG (115-035-003; Jackson). Protein markers (20351ES76; Shanghai Yisheng, China) were added on both sides of each gel to verify bands. The PVDF membranes were detected by enhanced che- moluminescence (Beyotime, China). Bands were analyzed using Image Lab™ Software (Bio-Rab Laboratories), were normalized to β-Tubulin and expressed as relative units (RU).

### Histopathology

Ovaries were fixed in PFA overnight with paraffin embedded, cut in 7-μm-thick sections, and stained with hematoxylin–eosin (H&E) using standard techniques.

### Immunohistochemistry

After deparaffinization in xylene and rehydrated through descending concentrations of ethanol, the slides were placed into sodium citrate buffer to recover antigen in a microwave oven. After incubating in 1% H2O2 for 10 min to inhibit endogenous peroxidases and blocking by using 5% bovine serum albumin for 30 min at 37 °C, the sections were incubated in a rabbit anti-Kiss1 polyclonal primary antibody(Solarbio, K009431P) and anti-Kiss1R polyclonal primary antibody (Solarbio, K003544P) at 4 °C overnight. Then, the sections identified using biotinylated goat anti-mice immune globulin G (1:3000; Jackson ImmunoResearch, USA). Sections were then incubated with ABC reagents (PK-6100; Vector Labs, USA) for 60 min. Immunoreactive sites were subsequently identified by 3,3 diaminobenzidine (DAB substrate kit; Vector Labs, USA), and tissue sections were photographed using a Nikon optical microscope (Nikon H600L). Semi-quantitative evaluation of the immunostaining intensity was carried out using Image-ProPlus 6.0 (IPP 6.0) system and mean optical density was used to represent the levels of protein expression [29, 30].

### Statistical analysis

Data were analyzed using GraphPad Prism 8 software (GraphPad Prism, Inc., San Diego, CA, USA). All physiological data were analyzed with unpaired t-test and parametric test, while all behavioral data were analyzed with t- test and nonparametric test. Results are presented as means ± SE, and the level of statistical significance was set at *p* < 0.05.

## Results

### Obesity and precocious puberty

**Fig. 1:**
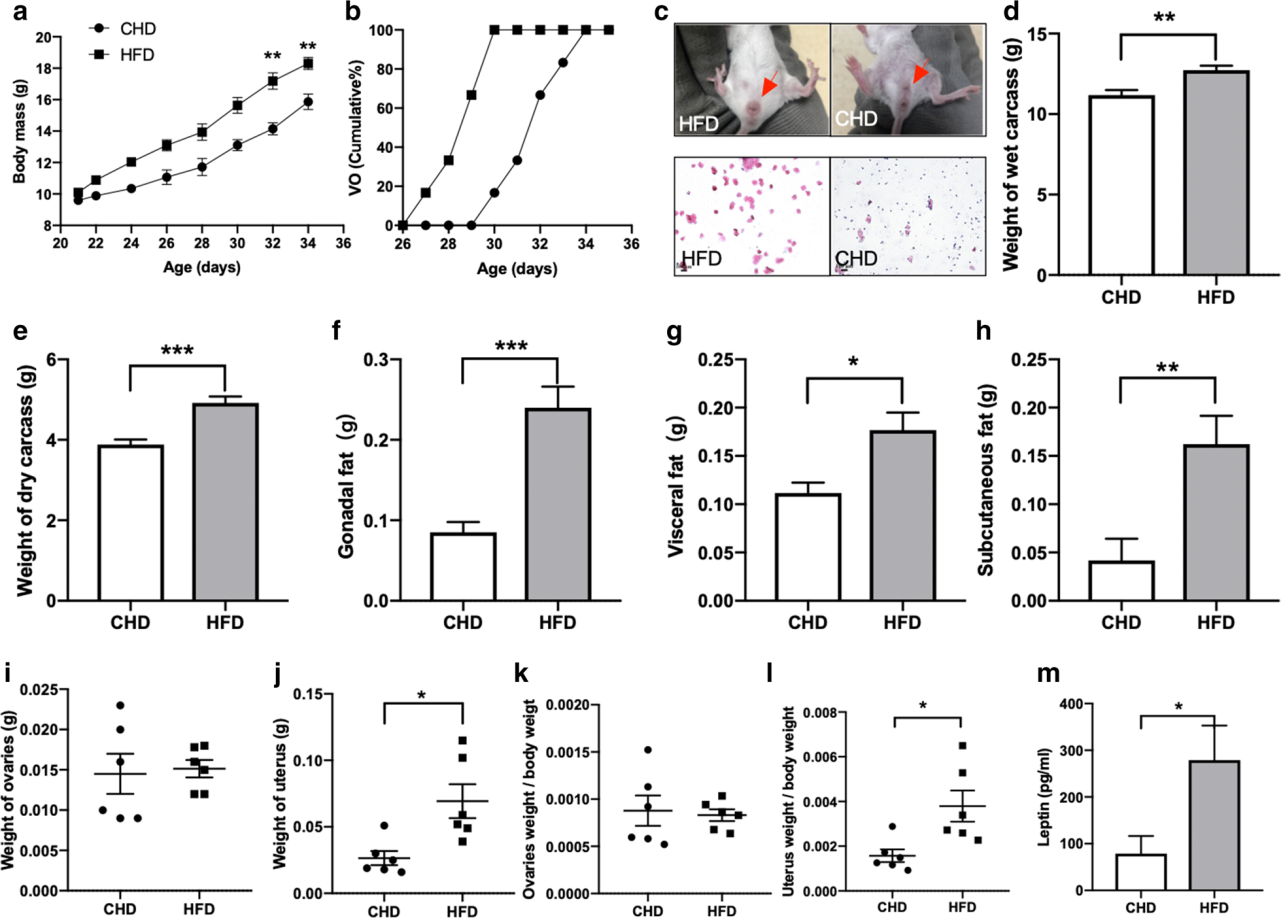
HFD leads to obesity and precocious puberty. **a** A marked increase in body mass in the HFD group compared with the CHD group. **b** Cumulative percentage of vaginal opening (VO). **c** Picture of Wight of vaginal opening and the vaginal exfoliated cell smear. **d**, **e** Weight of wet and dry carcass in HFD group were higher than CHD. **f**–**h** The weight of gonadal fat, visceral fat and subcutaneous fat in HFD group were higher than CHD group. **i**, **j** Weight of ovarian and uterus. **k**, **l** Ovaries weight/body weight and uterus weight/body weight in CHD and HFD groups. **m** Serum leptin concentration in CHD and HFD groups. Data are means ± SEM. *P < 0.05, **P < 0.01, and ***P < 0.001

To investigate the involvement of high fat diet in the precocious puberty linked to obesity, we fed weaned female mice with a high-fat diet. At baseline, all the 21 days old female mice exhibited similar body mass, and body mass over time was higher in the HFD mice than in the CHD mice (Fig. 1a). From the 32th day, there was significant difference in body mass between the two groups(t = 2.80, *p* = 0.0188, Fig. 1a). We used the vaginal opening as an indicator of puberty. In HFD group, vaginal opening appeared from 27 day-old, all mice reached puberty before 30 day-old. However, the puberty in CHD mice was late, and all of them reached puberty on the 35th day (Fig. 1b). At the same time, vaginal smear was used to determine the estrus period. There were obvious openings in the vagina of HFD mice, and CHD group were closed (Fig. 1c). The average age of puberty in the HFD group was 4 days earlier than CHD mice. The dry carcass and wet carcass in HFD group was higher than CHD (dry carcass: t = 5.049, *p* = 0.0005, wet carcass: t = 3.740, *p* = 0.0038, Fig. 1d, e). The subcutaneous fat, gonadal fat and visceral fat in HFD mice were 287.8%(t = 3.265, *p* = 0.0085), 181.8% (t = 5.316, *p* = 0.0003)and 58.3% (t = 3.114, *p* = 0.011)higher than CHD mice, respectively(Fig. 1f–h). There was no difference in the weight of ovaries between the two groups (Fig. 1i), but the weight of uterus was significantly higher in the HFD group (t = 3.095, *p* = 0.0114, Fig. 1j). The uterine index of HFD group was also significantly higher than CHD group (Fig. 1k), while the ovaries index was no difference(t = 2.945, *p* = 0.0147, Fig. 1l). Fat-derived adipokine leptin, an essential regulator of puberty was measured by serum. The results showed leptin in HFD mice was higher than CHD mice (t = 2.404, *p* = 0.0429, Fig. 1m).

### Hypothalamic- pituitary–gonadal axis

**Fig. 2:**
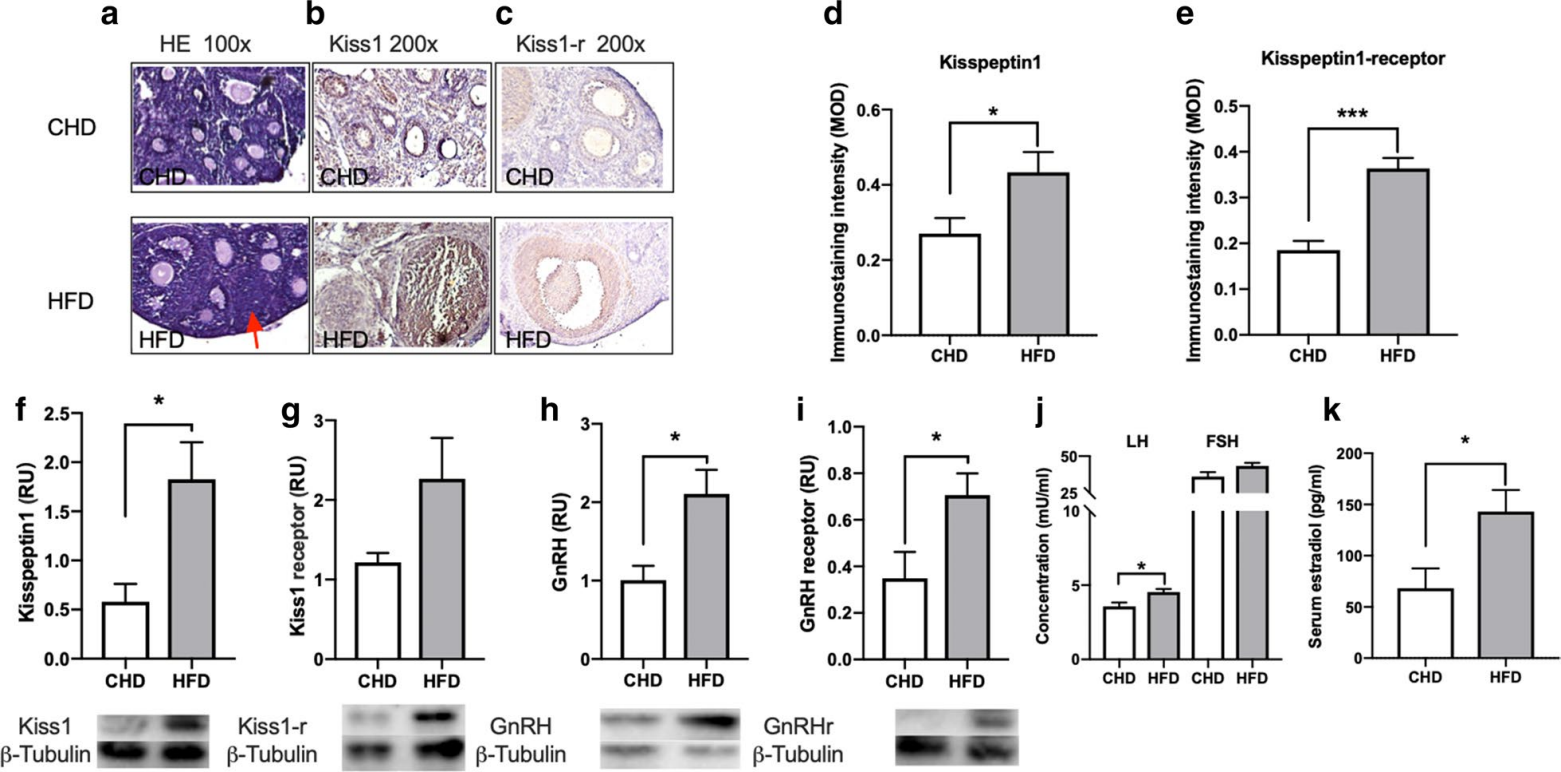
HFD feeding affects hypothalamic- pituitary–gonadal axis. **a** Effects of HFD after weaning in early follicular development(HE 100x). **b**, **c** Representative photomicrographs of kisspeptin1 and kisspeptin1 receptor immunostaining of ovaries. **d**, **e** MOD for kisspeptin1 and kisspeptin1 receptor were summarized. **f**, **g** Quantification of the levels of protein to kisspeptin1 and kisspeptin1 receptor in hypothalamus by western blotting. **h**–**i** Quantification of the levels of protein to GnRH and GnRH receptor in hypothalamus by western blotting. **j** Serum LH and FSH in two groups. **k** Serum estradiol was increased in HFD group. Data are means ± SEM. *P < 0.05, **P < 0.01, and ***P < 0.001

To analyze follicular development during the prepubertal stage, we studied the ovaries of CHD and HFD mice by morphometry and related it with some mRNA expression levels. Figure 2a showed the representative photomicrographs of ovaries from CHD and HFD (100x). In HFD group, follicles were larger and corpus luteum appeared (red arrow), which was the characteristic of ovarian maturation. The ovary of the HFD mice had a higher mRNA expression level of Kisspeptin1(t = 2.384, *p* = 0.0383) and Kisspeptin1-receptor (t = 5.915, *p* = 0.0001) than the ovary of CHD mice (Fig. 2d, e). The protein expression of hypothalamus Kisspeptin1 and Kisspeptin1-receptor was analyzed. The results showed distinctly increased expression of Kisspeptin1 of the HFD mice compared with the CHD mice (t = 2.982, *p* = 0.0138), while there was no significant difference in the Kisspeptin1-receptor expression between two groups (Fig. 2f, g). Also, the expression of GnRH (t = 3.084, *p* = 0.015) and GnRH receptor (t = 2.426, *p* = 0.0357) increased in the HFD mice compared with the CHD mice (Fig. 2h, i). The serum LH and FSH concentration were measured by ELISA, and LH level was higher in HFD mice than CHD mice, while FSH was no difference (LH: t = 2.845, *p* = 0.017, FSH: t = 1992, *p* = 0.074, Fig. 2j). Finally, we measured estradiol content of serum, HFD mice had a higher level than CHD mice (t = 2.622, *p* = 0.0255, Fig. 2k).

### Results of behaviors and neuroproteins

**Fig. 3:**
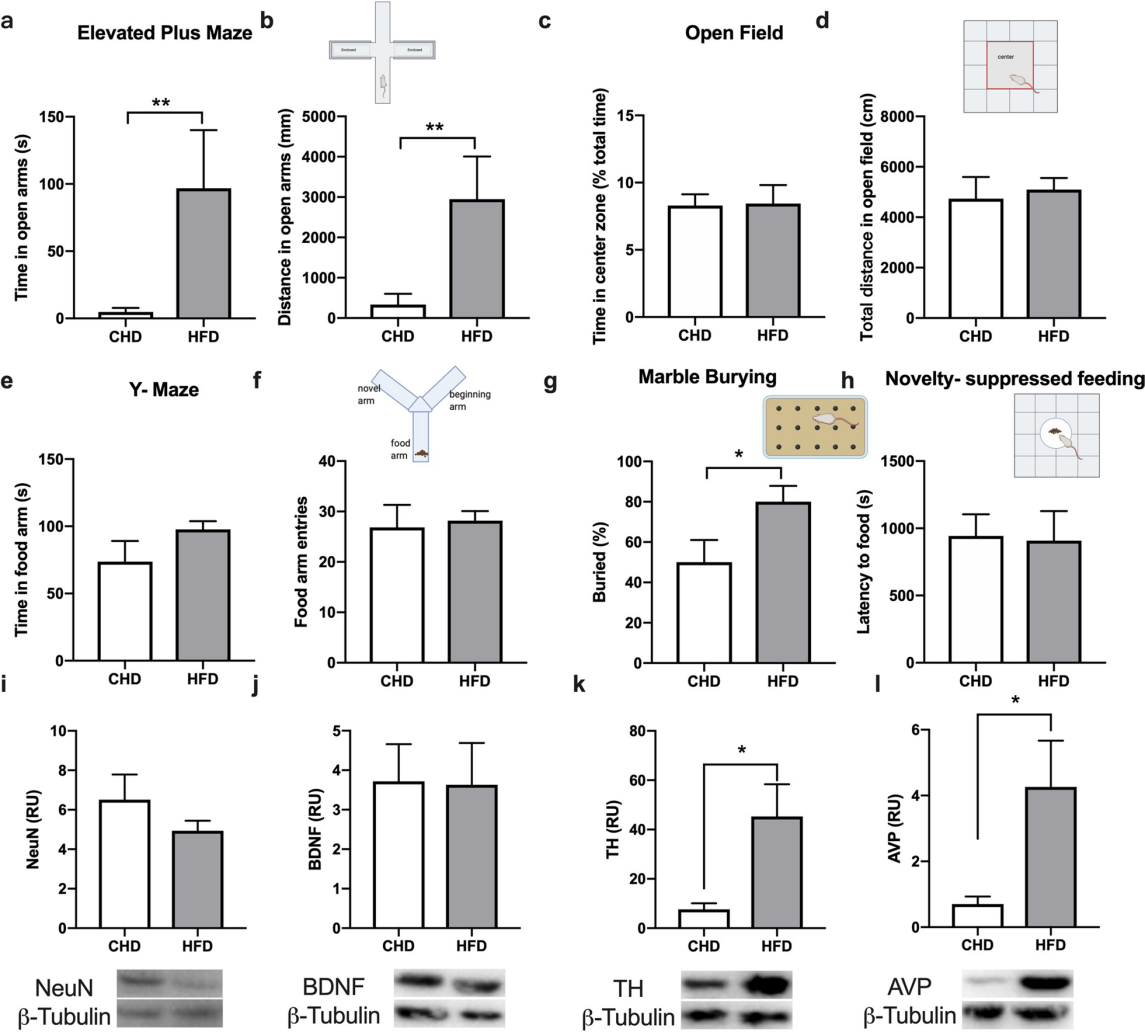
High-fat diet after weaning increases anxiety and risk taking behavior. **a**, **b** Time spent exploring and distance in the open arms of the elevated plus maze was significantly increased in HFD as compared with CHD mice. **c**, **d** Time spent and total distance traveled in the inner zone of the open field were no significantly difference in HFD mice and CHD mice. **e**, **f** Time in food arm and food arm entries were no significantly difference in HFD mice and CHD mice in y-maze test. **g** Marble-burying behavior was significantly increased in HFD versus HFD mice. **h** Novelty-suppressed feeding was no significantly difference. **i**–**l** Quantification of the levels of protein to NeuN, BDNF, TH and AVP in hypothalamus by western blotting. Data are means ± SEM. *P < 0.05, **P < 0.01, and ***P < 0.001

Risk-taking behavior assessed using the elevated plus maze. Mice with strong exploratory and risk-taking are more inclined to the open arm. Results showed the distance in the open arms of HFD mice was higher than CHD mice (t = 2.398, *p* = 0.0375, Fig. 3b), which means HFD mice were more risk-taking. In the open field experiment, the mice with strong exploratory preferred the central area, while there was no difference of the time and distance spent in the center area both in HFD mice and CHD mice (Fig. 3c, d). In the Y-maze test, the mice with strong memory preferred the food arm, however, the time in food arm and entries to food arm were no difference between two groups (Fig. 3e, f). Marble burying test measures the anxiety of mice by digging behavior [25], because anxious mice bury more marbles. HFD mice displayed a 1.5-fold increase in marble burying compared to CHD mice (t = 2.216, *p* = 0.05, Fig. 3g). Finally, to further assess anxiety, mice were subjected to a novelty-suppressed feeding test, in which fasted mice are placed in an open-field, and a white plate containing food pellets in its center, which creates anxiety to enter the center and get food. As a result, there was no difference between two groups in the latency to food (Fig. 3h). In order to explain anxiety and risk-taking behavior, we measured the expression of various neuroproteins in hypothalamus. The content of NeuN and BDNF in hypothalamus were no different between two groups (Fig. 3i, j), while the TH (t = 2.834, *p* = 0.0177) and AVP (t = 2.50, *p* = 0.0315) were higher in HFD group than CHD group (Fig. 3k, l).

### Results of estradiol treatment experiment

**Fig. 4:**
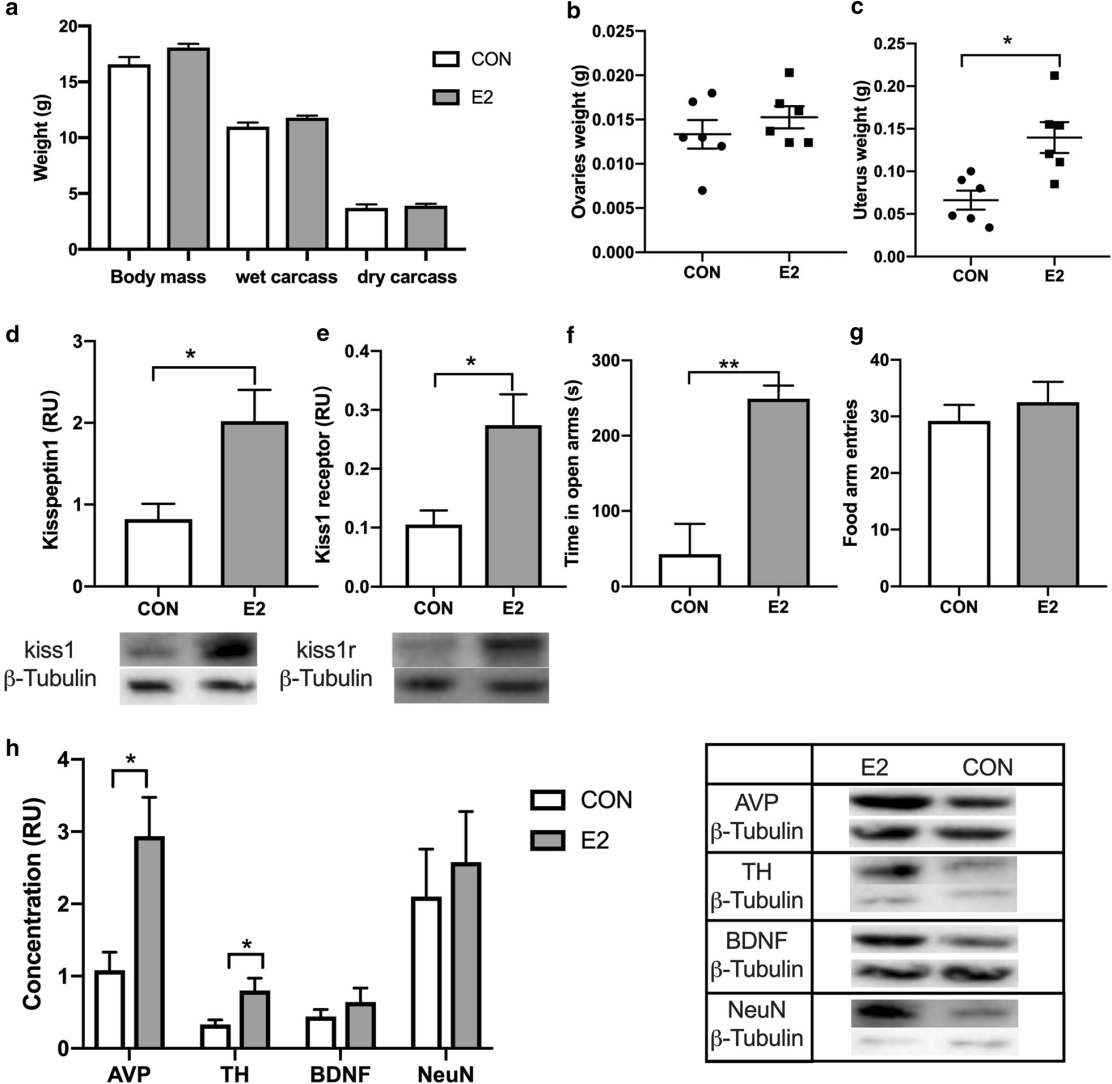
Effects of estradiol on enter puberty and neurodevelopment in mice. **a** Body weight, wet carcass and dry carcass weights in E2 and CON group. **b**, **c** The weight of ovaries was no difference, while uterus weight was higher in E2 group than CON group. **d** Time spent in the open arms of the elevated plus maze was significantly increased in E2 as compared with CON mice. **e** Entries of food arm was increased of E2 than CON group in y-maze test. **f**, **g** Quantification of the levels of protein to kisspeptin1 and kisspeptin1 receptor in hypothalamus by western blotting. **h** Quantification of the levels of protein to NeuN, BDNF, TH and AVP in hypothalamus by western blotting, TH and AVP were higher in E2 group. Data are means ± SEM. *P < 0.05, **P < 0.01, and ***P < 0.001

To verify the relationship between precocious puberty and adolescent behavior, we used estradiol treatment to create a model of precocious puberty. We found that, unlike high-fat diet, estradiol had no effect on body weight (Fig. 4a). The weight of uterus increased significantly in E2 group (t = 3.448, *p* = 0.0062), but the ovaries did not change (Fig. 4b, c). The protein expression of Kisspeptin1 (t = 2.819, *p* = 0.0182) and Kisspeptin1-receptor (t = 2.938, *p* = 0.0148) in hypothalamus showed distinctly increased of the E2 mice compared with the control mice (Fig. 4d, e). In the behavioral test, the risk-taking behavior of group E2 was significantly increased, and they spent more time in the open arm (t = 4.717, *p* = 0.0008, Fig. 4f). In the Y-maze, entries to food arm were no significantly difference, indicating that hormone induced-precocious puberty did not damage the memory of mice (Fig. 4g). Finally, from the results of neurotransmitter, AVP (t = 3.141, *p* = 0.0105) and TH (t = 2.609, *p* = 0.0261)were significantly increased in E2 group than CON group(Fig. 4h).

### Results of behaviors and neuroproteins in adults

**Fig. 5:**
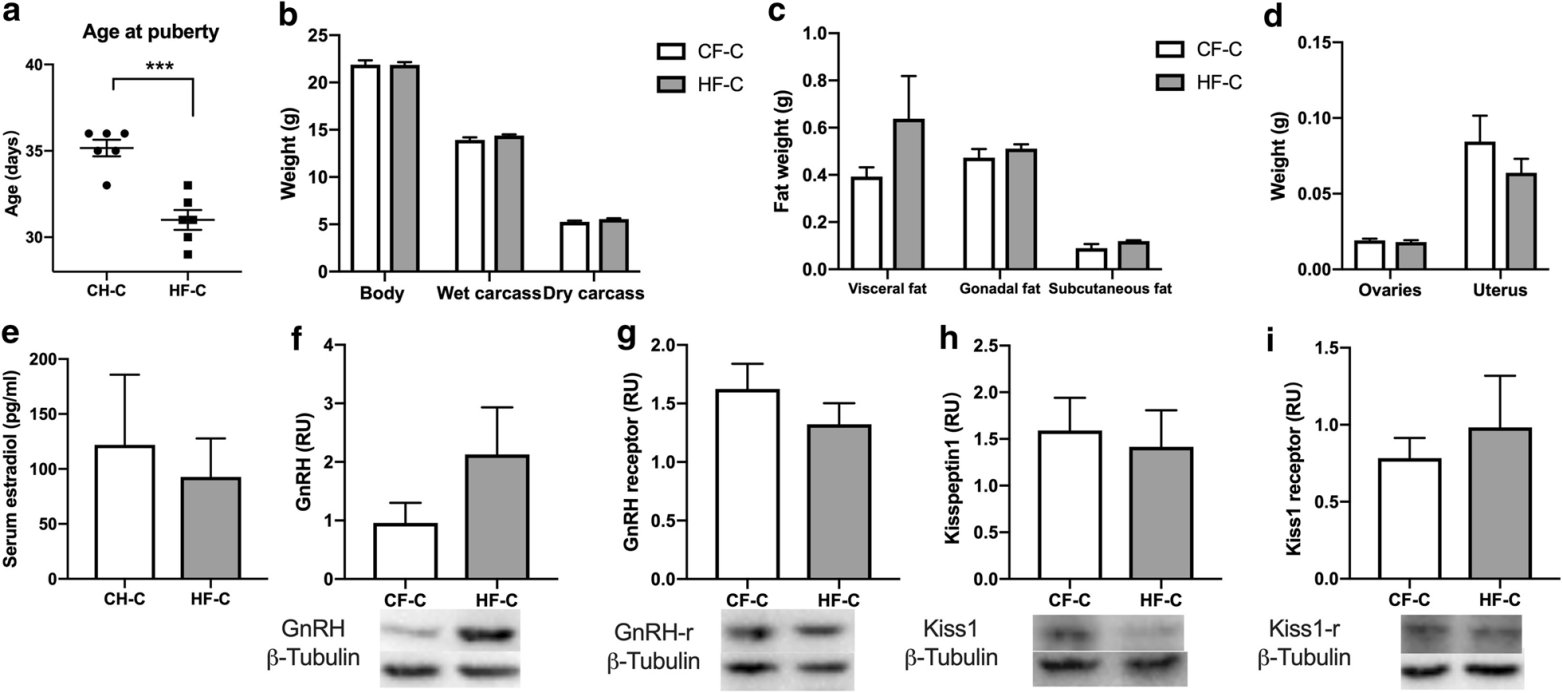
Comparison of energy metabolism and neurodevelopment between HF-C and CH-C groups. **a** Cumulative percentage of vaginal opening (VO). **b** Body weight, wet carcass and dry carcass weights in HF-C and CH-C group. **c** The weight of subcutaneous fat, gonadal fat and visceral fat in HFD and CHD groups. **d** The weight of ovaries and uterus were no difference between two groups. **e** Diet had no significant effects on the levels of serum estradiol. **f**, **g** Quantification of the levels of protein to GnRH and GnRH receptor in hypothalamus by western blotting. **h**, **i** Quantification of the levels of protein to kisspeptin1 and kisspeptin1 receptor in hypothalamus by western blotting. Data are means ± SEM. *P < 0.05, **P < 0.01, and ***P < 0.001

High fat diet made mice reach puberty earlier (t = 5.562, *p* = 0.0002, Fig. 5a), but after changed to normal diet, its obesity would disappear in adult (Fig. 5b). During adulthood, subcutaneous fat, gonadal fat and visceral fat were no difference between two groups (Fig. 5c). We also found there was no difference in the weight of ovaries and uterus between the two groups (Fig. 5d). The estradiol content of serum were no difference (Fig. 5e). The protein content of hypothalamus Kisspeptin1 and Kisspeptin1-receptor were no difference, also the protein content of GnRH and GnRH receptor in the two groups were no difference (Fig. 5f, i).

**Fig. 6:**
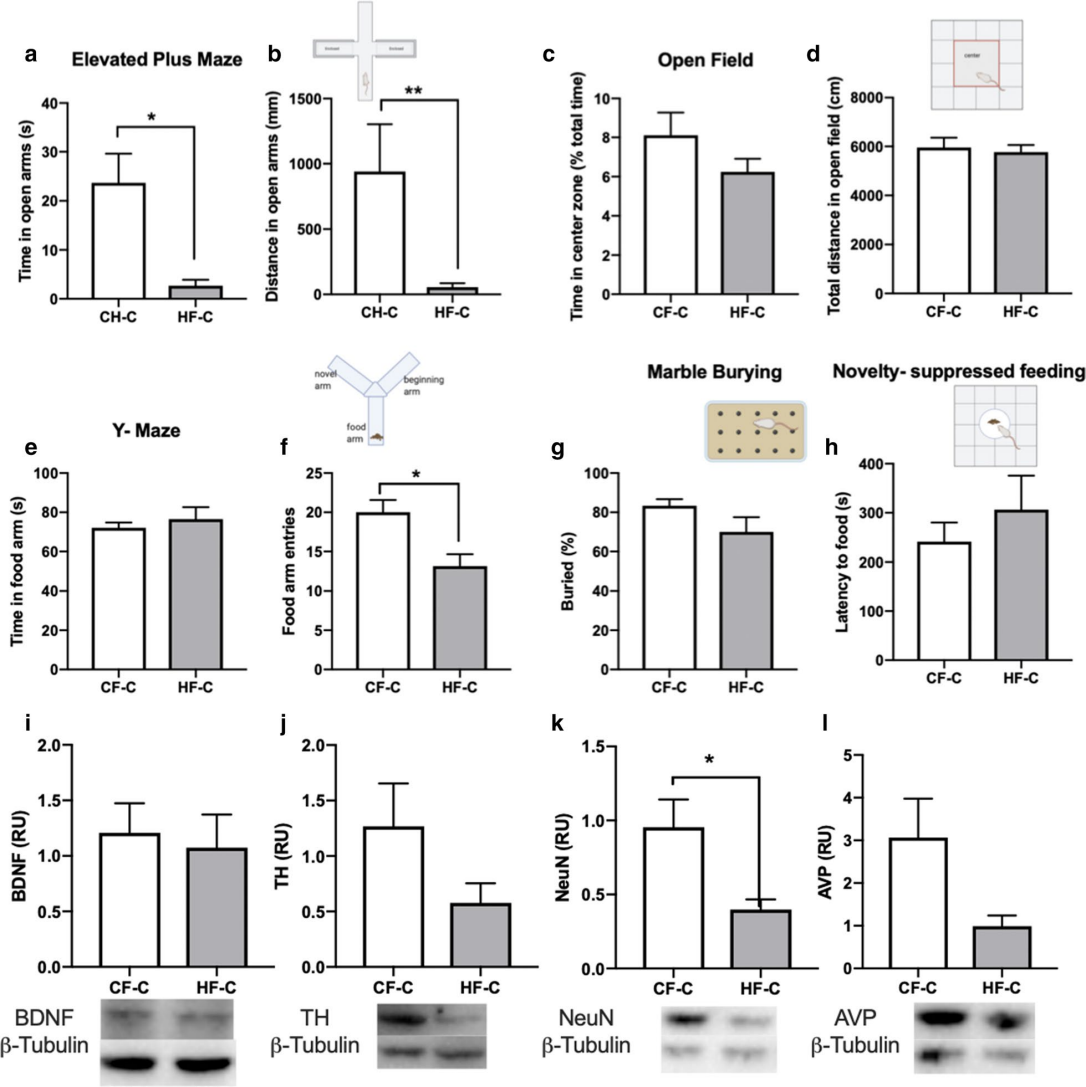
High-fat diet in childhood increases anxiety and decreases memory when they reach young adulthood. **a**, **b** Time spent exploring and distance in the open arms of the elevated plus maze was significantly decreased in HF-C as compared with CH-C mice. **c**, **d** Time spent and total distance traveled in the inner zone of the open field were no significantly difference in two groups. **e**, **f** Time in food arm was no significantly difference but food arm entries of HF-C was lower than CF-C group in y-maze test. **g** Marble-burying behavior was no significantly difference in two groups. **h** Novelty-suppressed feeding was significantly difference in two groups. **i**–**l** Quantification of the levels of protein to NeuN, BDNF, TH and AVP in hypothalamus by western blotting. Data are means ± SEM. *P < 0.05, **P < 0.01, and ***P < 0.001

From the elevated plus maze, we found that HF-C mice spent significantly less time (t = 3.810, *p* = 0.0042) and less distance (t = 2.685, *p* = 0.025) in the open arms of the maze in adulthood (Fig. 6a, b). For open field test, the total distance in open field was no difference between two groups, though there was a downward trend in the time in center zone of HF-C mice (Fig. 6c, d). However, in Y-maze test, the entries to food arm in HF-C mice was significantly lower than CF-C mice (t = 3.154, *p* = 0.0103, Fig. 6e, f), while the time in food arm was no different. There was no difference between CF-C and HF-C mice in the results of the marble burying test (Fig. 6g). For the novelty-suppressed feeding, there was no difference between two groups in the latency to food (Fig. 6h). We investigated changes in concentrations of neurotransmitters which have been associated with social behavior and cognition. Mice with HFD feeding in the childhood had a low level of NeuN (t = 2.782, *p* = 0.0194, Fig. 6i–l) in adulthood.

## Discussion

This study found that high-fat diet after weaning can cause precocious puberty in mice, and affect their behavior and cognition of puberty, like increase their risk-taking behavior and exploration ability. However, when HFD-induced precocious puberty mice changed to normal diet, their body fat and other physiological indicators would gradually recover, but it would increase anxiety and memory decline in adulthood. The content of NeuN in hypothalamus of HFD mice decreased in adulthood, which indicated that HFD-precocious puberty has a negative effect on nerve development.

### High fat diet in childhood leads to obesity and precocious puberty

Obesity caused by changes in diet and lifestyle is becoming an epidemic. Obesity is associated with a variety of complications, such as diabetes, cardiovascular disease, metabolic disorders and reproductive problems [31]. Obesity can affect reproductive function [32–34], such as earlier puberty in women [35]. Recent studies have shown that early-onset obesity increases the content of ceramide in hypothalamus and promotes puberty in rats [36]. Our results showed that high-fat diet induced weight gain and white adipose tissue disposition significantly increased (including visceral fat, subcutaneous fat and gonadal fat) in HFD group. However, the changing of diet (CHD was used after puberty) can make the weight and fat content of obese mice return to normal level in adulthood.

In this study, we found that HFD mice reached puberty earlier, and our results are consistent with previous studies [36]. It has been proved that the central kisspeptin / GPR54 system is the key player to regulate puberty onset by stimulating HPG axis [37, 38]. Hypothalamic KiSS1 neurons are involved in the regulation of positive and negative feedback effects of estrogen [39]. Previous studies have shown that the expression of kisspeptin is preferentially increased in the hypothalamus in the over nourished animal models that lead to precocious puberty [40, 41]. At the same time, maternal HFD during pregnancy impaired the early follicular development of newborns, which may be related to kisspeptin / GPR54 system [29, 30]. We also found HFD in childhood can increase the expression of kisspeptin in the ovaries of mice, leading to the puberty. We detected the content of kisspeptin/kisspeptin receptor and GnRH / GnRH receptor in hypothalamus, and found that HFD group was higher than CHD group. Also, the LH and E2 were higher in HFD mice. Therefore, the precocious puberty induced by high-fat food in childhood is mediated by kisspeptin -GnRH-LH-E2 axis.

### Precocious puberty increases risk-taking behavior and adult anxiety behavior

Age has a great influence on the behavior of mice. For example, young and adult mice strongly avoid entering the open arm. In contrast, adolescents enter the open arm faster and more frequently than other age groups [16]. This shows that in this adolescence, the level of pursuit of novelty increased, more risk-taking behavior, and the behavior and physiological response to stressful situations decreased. Additionally, the effect of age on behavior is related to sex hormone, for example progesterone was related to individual exploration and risk taking [17]. Here, we use the elevated maze to test animals' risk-taking behavior and stress response. Consistent with previous evidence, we found that HFD mice had a higher rate of open arm entry than CHD mice. Therefore, we suggest that HFD-induced precocious puberty mice exhibit lower anxiety related responses and higher risk-taking behaviors. At the same time, in Experiment 2, the risk-taking behavior of precocious puberty mice induced by estrogen was also increased. This also confirmed that high-fat diet made mice enter puberty earlier, not only physiological changes, but also behavioral changes. In the open field and new food recognition experiments, high-fat feeding did not increase the level of animal anxiety. Only in the test of burying behavior, mice in HFD group showed a higher degree of buried in bead embedding test. This may be related to the increase of curiosity about new things, but it is also the reason for anxiety. Although a large number of studies have confirmed that high-fat diet can increase animal anxiety [42], for precocious mice, the pursuit of new things and risk-taking behavior are the main factors to enter the open arm.

However, our experiments also found that high-fat feeding experiences in infancy made adult mice more anxious when they returned to normal diet. For example, they visited the open arms less often or even not in the elevated cross maze. At the same time, in the Y-maze test, the number of visits to the food arm decreased, suggesting that their memory was impaired. Overall, these data are consistent with a large number of researches that describing the brain's sensitivity to a high-fat diet [43, 44]. It can be seen that precocious puberty caused by high-fat in adolescence may have a lifelong and irreversible negative impact on brain nerve development.

### Precocious puberty and behavioral changes induced by high-fat food are related to the neurodevelopment

Both humans and mice are more likely to take risks in adolescence, which is related to the competition between social emotional system and cognitive system in the brain. In early adolescence, dopamine system in prefrontal cortex is active, which plays an important role in brain reward cycle. Therefore, risk-taking behavior of adolescent mice will increase [45]. High fat food, can affect the development of the brain, but also affect the behavior of animals. Some studies have shown that the effect of high-fat diet during pregnancy on the brain development of offspring may be reflected in the increase of dopamine in nucleus accumbens, the decrease of endogenous cannabinoid in hippocampus structure and the length of amygdala dendrite, which may lead to the increase of aggressive behavior and depression behavior of offspring [46]. Similarly, when the adolescent male rats were fed with high-fat food, their anxiety like behavior was enhanced and the structure and neural circuits of related brain regions were changed [42]. The effects of high-fat diet during pregnancy and adolescence on animal behavior and brain development were studied. Our experiments focused on the effects of high-fat foods on mice during childhood, from post weaning to pre puberty. Recent studies have shown that diet induced obesity in mice results in impaired hippocampal memory, but there is no significant change in BDNF [47], which is consistent with our results. There is no difference in BDNF between HFD group and control group. However, in our results, AVP of HFD-induced precocious mice and E2-induced precocious mice were higher than those in the control group, which could explain the risk-taking behavior and exploratory increase [48]. Previous studies have shown that long-term HFD during the critical period of neural development (puberty) may cause some irreversible changes in brain and behavior [42]. For example, in young animals, HFD can affect brain development through neurogenesis and affect cognitive ability in adulthood [49]. As our results, HFD- induced precocious puberty mice have memory impairment in adulthood, and TH, NeuN, AVP in hypothalamus related to cognition and memory are significantly reduced. Combined with the results of several behavioral tests of the two groups of animals, we believe that HFD induced precocious puberty can cause irreparable damage to the brain of adult mice, such as memory impairment and increase the anxiety of animals. Therefore, HFD- induced precocious puberty will not only increase the risk-taking behavior in adolescence, but also damage the development of brain nerve during the growth of adolescents, and affect the cognitive phenotype of adults.

## Conclusions

High fat diet after weaning can cause the precocious puberty, increase the risk-taking behavior of mice, and affect the neurodevelopment. During adulthood, the body weight, body fat content and other metabolic index of mice recovered to normal level by chow diet, but the effect on neurodevelopment was not completely recovered, anxiety and memory impairment were increased in adult mice. Therefore, we can not ignore the harm of HFD on precocious puberty. Our results also have implications for human studies. We suggest that reduce the excessive intake of high-fat food in childhood, because obesity induced precocious puberty may lead to lifelong damage.

## Data Availability

The data that support the findings of this study are available from the corresponding author upon reasonable request.

## Abbreviations

HFD: High fat diet
H&E: Hematoxylin–eosin
EPM: Elevated plus maze
HPG: Hypothalamus-pituitary–gonadal
GnRH: Gonadotropin-releasing hormone
GnRHR: Gonadotropin-releasing hormone receptor
Kiss1: Kisspeptin
Kiss1R: Kisspeptin1 receptor
TH: Tyrosine hydrogenase
AVP: Arginine vasopressin
NeuN: Neuronal nuclei
BDNF: Brain-derived neurotrophic factor

## References

[1] Parent AS, Teilmann G, Juul A, Skakkebaek NE, Toppari J, Bourguignon JP (2003). The timing of normal puberty and the age limits of sexual precocity, variations around the world, secular trends, and changes after migration. Endocr Rev.

[2] Kalantaridou SN (2002). Monogenic disorders of puberty. J Clin Endocrinol Metab.

[3] Lakshman R, Forouhi N, Luben R, Bingham S, Khaw K, Wareham N, Ong KK (2008). Association between age at menarche and risk of diabetes in adults, results from the EPIC-Norfolk cohort study. Diabetologia.

[4] Ritte R, Tikk K, Lukanova A, Tjønneland A, Olsen A, Overvad K, Dossus L, Fournier A, Clavel-Chapelon F, Grote V (2013). Reproductive factors and risk of hormone receptor positive and negative breast cancer, a cohort study. BMC Cancer.

[5] Golub MS, Collman GW, Foster PMD, Kimmel CA, Rajpert-De Meyts E, Reiter EO, Sharpe RM, Skakkebaek NE, Toppari J (2008). Public health implications of altered puberty timing. Pediatrics.

[6] Miguel A, Sanchez-Garrido MT (2013). Metabolic control of puberty, roles of leptin and kisspeptins. Horm Behav.

[7] De Leonibus C, Marcovecchio ML, Chiavaroli V, de Giorgis T, Chiarelli F, Mohn A (2014). Timing of puberty and physical growth in obese children, a longitudinal study in boys and girls. Pediatr Obes.

[8] Aksglaede L, Juul A, Olsen LW, Sørensen TIA, Tena-Sempere M (2009). Age at puberty and the emerging obesity epidemic. PLoS ONE.

[9] Castellano JM, Tena-Sempere M (2016). Metabolic control of female puberty, potential therapeutic targets. Expert Opin Ther Tar.

[10] Chehab FF (2014). Leptin and reproduction, past milestones, present undertakings, and future endeavors. J Endocrinol.

[11] Hrabovszky E, Ciofi P, Vida B, Horvath MC, Keller E, Caraty A, Bloom SR, Ghatei MA, Dhillo WS, Liposits Z, Kallo I (2010). The kisspeptin system of the human hypothalamus, sexual dimorphism and relationship with gonadotropin-releasing hormone and neurokinin B neurons. Eur J Neurosci.

[12] Ohtaki T, Shintani Y, Honda S, Hirokazu M, Akira H, Kimiko K, Yasuko T, Satoshi K, Yoshihiro T, Yasushi M (2001). Metastasis suppressor gene KiSS-1 encodes peptide ligand of a G-protein-coupled receptor. Nature.

[13] Wahab F, Atika B, Shahab M, Behr R (2016). Kisspeptin signalling in the physiology and pathophysiology of the urogenital system. Nat Rev Urol.

[14] Teles MG, Tusset C, Latronico AC (2011). New genetic factors implicated in human GnRH-dependent precocious puberty, the role of kisspeptin system. Mol Cell Endocrinol.

[15] Macr S, Adriani W, Chiarotti F, Laviolaf G (2002). Risk taking during exploration of a plus-maze is greater in adolescent than in juvenile or adult mice. Anim Behav.

[16] Magnus L, Inga-Maj J, Bengt M, Per L, Torbjörn B (2006). Progesterone withdrawal effects in the open field test can be predicted by elevated plus maze performance. Horm Behav.

[17] Ribes-Navarro A, Atef M, Sánchez-Sarasúa S, Beltrán-Bretones MT, Olucha-Bordonau F, Sánchez-Pérez AM (2019). Abscisic acid supplementation rescues high fat diet-induced alterations in hippocampal inflammation and IRSs expression. Mol Neurobiol.

[18] McLean FH, Grant C, Morris AC, Horgan GW, Polanski AJ, Allan K, Campbell FM, Langston RF, Williams LM (2018). Rapid and reversible impairment of episodic memory by a high-fat diet in mice. Sci Rep.

[19] Ullah R, Su Y, Shen Y, Li CL, Xu XQ, Zhang JW, Huang K, Rauf N, He Y, Cheng JJ, Qin HP, Zhou YD, Fu JF (2017). Postnatal feeding with high-fat diet induces obesity and precocious puberty in C57BL/6J mouse pups, a novel model of obesity and puberty. Front Med.

[20] Bo TB, Zhang XY, Kohl KD, Wen J, Tian SJ, Wang DH (2020). Coprophagy prevention alters microbiome, metabolism, neurochemistry and cognitive behaviour in a small mammal. J ISME.

[21] Lofgren M, Johansson I, Meyerson B, Turkmen S, Bäckström T (2009). Withdrawal effects from progesterone and estradiol relate to individual risk-taking and explorative behavior in female rats. Physiol Behav.

[22] Sharma S, Fulton S (2013). Diet-induced obesity promotes depressive-like behaviour that is associated with neural adaptations in brain reward circuitry. Int J Obes.

[23] Snyder CN, Brown AR, Buffalari D (2020). Similar tests of anxiety-like behavior yield different results: comparison of the open field and free exploratory rodent procedures. Physiol Behav.

[24] Njung’e K., Handley, S.L. (1991). Evaluation of marble-burying behavior as a model of anxiety. Pharmacol Biochem Behav.

[25] Snyder JS, Soumier A, Brewer M, Pickel J, Cameron HA (2011). Adult hippocampal neurogenesis buffers stress responses and depressive behaviour. Nature.

[26] Lin S, Tian L (2015). Temporal dynamics of anxiety phenotypes in a dental pulp injury model. Mol Pain.

[27] Li XS, Wang DH (2007). Photoperiod and temperature can regulate body mass, serum leptin concentration, and uncoupling protein 1 in Brandt’s voles (*Lasiopodomys brandtii*) and Mongolian gerbils (*Meriones unguiculatus*). Physiol Biochem Zool.

[28] Zhou Z, Lin Q, Xu X, Illahi GS, Dong C, Wu X (2019). Maternal high-fat diet impairs follicular development of offspring through intraovarian kisspeptin/gpr54 system. Reprod Biol Endocrin.

[29] Zhou Z, Lin Q, Xu X, Illahi GS, Dong CL, Wu XQ (2019). Maternal high-fat diet impairs follicular development of offspring through intraovarian kisspeptin/GPR54 system. Reprod Biol Endocrinol.

[30] Mayes J, Watson G (2004). Direct effects of sex steroid hormones on adipose tissues and obesity. Obes Rev.

[31] Colmenares A, Gunczler P, Lanes R (2014). Higher prevalence of obesity and overweight without an adverse metabolic profile in girls with central precocious puberty compared to girls with early puberty, regardless of GnRH analogue treatment. Int J Pediatr Endocrinol.

[32] Hussain MA, Abogresha NM, Hassan R, Tamany DA, Lotfy M (2016). Effect of feeding a high-fat diet independently of caloric intake on reproductive function in diet-induced obese female rats. Arch Med Sci.

[33] Pasquali R, Pelusi C, Genghini S, Cacciari M, Gambineri A (2003). Obesity and reproductive disorders in women. Hum Reprod Update.

[34] Dai YL, Fu JF, Liang L, Gong CX, Xiong F, Luo FH, Liu GL, Chen SK (2014). Association between obesity and sexual maturation in Chinese children, a muticenter study. Int J Obes.

[35] Heras V, Castellano JM, Fernandois D, Velasco I, Tena-Sempere M (2020). Central ceramide signaling mediates obesity-induced precocious puberty. Cell Metabol.

[36] Matsui H, Takatsu Y, Kumano S, Matsumoto H, Ohtaki T (2004). Peripheral administration of metastin induces marked gonadotropin release and ovulation in the rat. Biochem Biophys Res Commun.

[37] Seminara SB, Messager S, Chatzidaki EE, Thresher RR, Acierno JS, Shagoury JK, Bo-Abbas Y, Kuohung W, Schwinof KM, Hendrick AG (2003). The GPR54 gene as a regulator of puberty. N Engl J Med.

[38] Smith JT, Cunningham MJ, Rissman EF, Clifton DK, Steiner RA (2005). Regulation of Kiss1 gene expression in the brain of the female mouse. Endocrinol.

[39] Castellano JM, Bentsen AH, Sánchez-Garrido MA, Ruiz-Pino F, Romero M, Garcia-Galiano D, Aguilar E, Pinilla L, Diéguez C, Mikkelsen JD, Tena-Sempere M (2011). Early metabolic programming of puberty onset, impact of changes in postnatal feeding and rearing conditions on the timing of puberty and development of the hypothalamic kisspeptin system. Endocrinol.

[40] Maria EK, Lie AO, Jens DM (2013). Effect of a postnatal high-fat diet exposure on puberty onset, estrous cycle regularity, and kisspeptin expression in female rats. Reprod Biol.

[41] Vega-Torres JD, Haddad E, Lee JB, Kalyan-Masih P, George WIM, Pérez LL, Vázquez DMP, Torres YA, Santana JMS, Obenaus A, Figueroa JD (2018). Exposure to an obesogenic diet during adolescence leads to abnormal maturation of neural and behavioral substrates underpinning fear and anxiety. Brain Behavi Immun.

[42] Freeman LR, Zhang L, Nair A, Dasuri K, Francis J, Fernandez-Kim SO, Bruce-Keller AJ, Keller JN (2013). Obesity increases cerebrocortical reactive oxygen species and impairs brain function. Free Radic Biol Med.

[43] Stranahan AM, Norman ED, Lee K, Cutler RG, Telljohann RS, Egan JM, Mattson MP (2008). Diet-induced insulin resistance impairs hippo- campal synaptic plasticity and cognition in middle-aged rats. Hippocampus.

[44] Sharma S, Fulton S (2011). Neurobehavioral adaptations to a rewarding high-fat and high-sugar diet in mice. Can J Diabetes.

[45] Gawlińska K, Gawliński D, Filip M, Przegaliński E (2020). Relationship of maternal high-fat diet during pregnancy and lactation to offspring health. Nutr Rev.

[46] Heyward FD, Walton RG, Carle MS, Coleman MA, Garvey WT, Sweatt JD (2012). Adult mice maintained on a high-fat diet exhibit object location memory deficits and reduced hippocampal SIRT1 gene expression. Neurobiol Learn Mem.

[47] Patel N, Grillon C, Pavletic N, Rosen D, Pine DS, Ernst M (2015). Oxytocin and vasopressin modulate risk-taking. Physiol Behav.

[48] Boitard C, Etchamendy N, Sauvant J, Aubert A, Tronel S, Marighetto A, Layé S, Ferreira G (2012). Juvenile, but not adult exposure to high-fat diet impairs relational memory and hippocampal neurogenesis in mice. Hippocampus.

[49] Pita J, Barrios V, Gavela-Pérez T (2011). Circulating kisspeptin levels exhibit sexual dimorphism in adults, are increased in obese prepubertal girls and do not suffer modifications in girls with idiopathic central precocious puberty. Peptides.

